# When Clades Collide: Genomic Admixture in Blacklegged Ticks (*Ixodes scapularis*) From the Great Plains

**DOI:** 10.1111/mec.70577

**Published:** 2026-10-07

**Authors:** Kaylee S. Herzog, Zach Pella, Halie Smith, Dayna McCormick, Lily Nuss, Amanda Brinkworth, Sujata S. Chaudhari, Kathryn Duncan, Kellee Sundstrom, Sydney Stein, Holly Black, Jose E. Pietri, Ryan C. Smith, Jeff Hamik, Roberto Cortiñas, Joseph R. Fauver

**Affiliations:** ^1^ Department of Biology and Museum of Southwestern Biology University of New Mexico Albuquerque New Mexico USA; ^2^ Department of Epidemiology University of Nebraska Medical Center Omaha Nebraska USA; ^3^ Association of Public Health Laboratories Bethesda Maryland USA; ^4^ Nebraska Department of Health and Human Services Lincoln Nebraska USA; ^5^ School of Veterinary Medicine and Biomedical Sciences University of Nebraska Lincoln Lincoln Nebraska USA; ^6^ Department of Pathology, Microbiology, and Immunology University of Nebraska Medical Center Omaha Nebraska USA; ^7^ Department of Veterinary Pathobiology Oklahoma State University Stillwater Oklahoma USA; ^8^ Department of Veterinary Clinical Medicine University of Illinois Urbana‐Champaign Illinois USA; ^9^ Department of Entomology Purdue University West Lafayette Indiana USA; ^10^ Department of Plant Pathology, Entomology and Microbiology Iowa State University Ames Iowa USA

## Abstract

*Ixodes scapularis* ticks transmit a number of pathogens important to human health, including *Borrelia burgdorferi* sensu stricto, the causative agent of Lyme disease. While *I. scapularis* is found across the Eastern United States, Lyme disease transmission is largely limited to the Northeast and Upper Midwest and is nearly absent in the South. This indicates that differences in northern versus southern clades of *I. scapularis* are associated with differences in Lyme disease transmission risk. *Ixodes scapularis* is undergoing range expansion, including into the Great Plains region of the United States. Determining where *I. scapularis* populations in the Great Plains originated can inform future risk of Lyme disease transmission in this region. In this study, we use a population genomics framework to characterize diversity, structure and *B. burgdorferi* infection rates of *I. scapularis* populations in the Great Plains region. We generated whole genome sequence data and single nucleotide polymorphism (SNP) datasets from *I. scapularis* ticks collected in Iowa, Kansas, Nebraska and South Dakota to compare to publicly available data from across the species' range. Our analysis of 200 *I. scapularis* SNP datasets indicated geographically well‐defined populations that correspond to historical northern and southern ancestral clades of *I. scapularis* . Ticks from South Dakota, Iowa and northeastern Nebraska are genetically similar populations from the Upper Midwest, while ticks from Kansas are more genetically similar to ticks from the Southeast. Ticks collected in the east central region of Nebraska, however, represent an evenly admixed population of both northern and southern genomic backgrounds. Analysis of *B. burgdorferi* reads from genomic datasets shows ~50% infection rate in ticks from Iowa and northeastern Nebraska, whereas ticks from Kansas show no evidence of *B. burgdorferi* infection. Of note, evenly admixed ticks from the east central region of Nebraska also show no evidence of *B. burgdorferi* infection. These results provide further evidence that tick genomics may influence traits associated with *B. burgdorferi* infection status, and thus the potential for Lyme disease transmission. As the range of *I. scapularis* continues to expand, bringing historically isolated populations into contact, there is a clear need to understand the consequence of genomic admixture for *B. burgdorferi* transmission potential to inform future risk of Lyme disease in the United States.

## 1 Introduction

*Ixodes scapularis* (blacklegged tick, deer tick) is an important vector of multiple pathogens relevant to human health, including *Anaplasma phagocytophilum, Babesia microti, Ehrlichia eauclairensis*, Powassan virus, *Borellia miyamotoi* and the causative agents of Lyme disease, *B. burgdorferi sensu stricto* and *B. mayonii* (Centers for Disease Control and Prevention n.d.). Lyme disease is the most prevalent vector‐borne disease (VBD) in the United States, and the annual number of diagnosed cases has been increasing for decades (Rosenberg et al. 2018; CDC 2025a). Unsurprisingly, the economic burden of Lyme disease in the United States is projected to be hundreds of millions of dollars annually (Hook et al. 2022). As with most VBDs, the reported case counts are likely substantial underestimates of total infections (Kugeler et al. 2021), thus making Lyme disease one of the most consequential infectious diseases in the United States. Lyme disease transmission is prevalent in the Northeast and Upper Midwest, but is nearly absent in the South (CDC 2025b), highlighting important geographical differences in risk.

The tick vector of *B. burgdorferi* , *I. scapularis* , is now distributed across much of the Eastern United States, but its demographic history is characterized by dramatic range expansion and contraction (Centers for Disease Control and Prevention n.d.; Eisen and Eisen 2023; Spielman 1994). Tick abundance decreased in response to deforestation and the collapse of white‐tailed deer populations ( *Odocoileus virginianus* ) across much of the Northern United States in the late 19th century. Following reforestation and recovery of deer populations, *I. scapularis* populations rebounded and expanded across the Northeast, Upper Midwest and Southeast (Eisen and Eisen 2023; Humphrey et al. 2010). Despite established populations across much of the Eastern United States, the burden of Lyme disease has largely been limited to the Northeast and Upper Midwest (Diuk‐Wasser et al. 2012; Pepin et al. 2012). Geographic differences in Lyme disease epidemiology can be attributed, in part, to regional differences in tick populations. Multiple studies have demonstrated *I. scapularis* populations have undergone isolation by distance and that gene flow is regionally restricted, resulting in genetically distinct northern and southern clades (Humphrey et al. 2010; Xu et al. 2020; Sakamoto et al. 2014; Van Zee et al. 2013; Schoville et al. 2024; Frederick et al. 2023). Life history traits in nymphal stage ticks important for *B. burgdorferi* transmission, such as questing behaviour and host preference, also differ by geography. Ticks collected in the Northeast and Upper Midwest quest above the leaf litter and prefer to feed on small mammals that maintain *B. burgdorferi* transmission cycles, whereas *I. scapularis* populations from the Southeast quest below the leaf litter and preferentially feed on reptiles, which are incompetent hosts for *B. burgdorferi* (Ginsberg et al. 2022; Arsnoe et al. 2019; Apperson et al. 1993; Tietjen et al. 2020; Durden et al. 2002; Spielman et al. 1979; Ogden et al. 2018). These life history traits result in differences in nymphal infection rates and ultimately, differences in human Lyme transmission risk (Diuk‐Wasser et al. 2012). Thus, contemporary *I. scapularis* populations collected in the Northeast and Upper Midwest are genetically and behaviourally distinct from ticks collected in the Southeast.

The range of *I. scapularis* is expanding into previously uninhabited regions, including the Great Plains. While populations of *I. scapularis* have been known for decades from Iowa (1989) (Novak et al. 1991) and Kansas (1988) (Coleman 2013), established populations have recently been identified in North Dakota (2010) (Russart et al. 2014), South Dakota (2015) (Maestas et al. 2016) and Nebraska (2018) (Nielsen et al. 2020), representing the western‐most edge of the species' distribution (Eisen and Eisen 2023). Additionally, *B. burgdorferi* has been detected in *I. scapularis* ticks from all three of these states (Russart et al. 2014; Black et al. 2021; Maestas et al. 2018; Hamik et al. 2023). Following the 2021 identification of autochthonous Lyme disease transmission in Nebraska, expanded tick surveillance identified two foci in northeastern and east central regions of the state. Molecular surveillance for *B. burgdorferi* in Nebraska demonstrates that *I. scapularis* from the northeastern region has a 55.4% prevalence in questing ticks (*n* = 119 tested), whereas ticks in the east central foci have a prevalence of 0% (*n* = 113 tested) (Nebraska Tick Surveillance: Blacklegged Tick n.d.). Clear differences in *B. burgdorferi* prevalence in questing ticks in Nebraska collected from geographically and ecologically similar areas led us to test the hypothesis that differences in *I. scapularis* population structure may be responsible for the observed difference in *B. burgdorferi* prevalence, indicating that both northern and southern populations of *I. scapularis* are present in the state.

In this study, we used whole genome sequencing (WGS) to characterize the genomic similarity, population structure and connectivity within and among recently‐established populations of *I. scapularis* in the Great Plains. We compared these newly‐generated datasets to WGS data from *I. scapularis* across the Eastern United States to better understand the genetic background of recently‐established populations. We also used WGS to explore the presence or absence of *B. burgdorferi* in individual ticks. Understanding the genetic ancestry of established ticks, how populations are structured and whether phenotypic differences exist between them can inform the risk of Lyme disease transmission in the Great Plains and other areas of recent *I. scapularis* range expansion.

## 2 Methods

### 2.1 Specimen Collection and Preservation

Sampling locations for ticks collected in this study are presented as open shapes in Figure 1, with sampling locations for publicly available datasets shown as closed shapes. Circles represent field collections and stars indicate that specimens were obtained from a laboratory colony. Adult *I. scapularis* ticks from recently established populations in South Dakota and Nebraska were prioritized for WGS. Additionally, samples collected from more established populations in Iowa and Kansas were sequenced to provide contextual data from populations of *I. scapularis* in close proximity and to add geographic contiguity among states previously sampled across the region. Detailed metadata for all specimens included in analyses, including sampling locality, life‐cycle stage, nucleic acid isolation methodology, sequencing and alignment summary statistics, etc. are presented in Table S1. Host‐seeking ticks were collected in eastern Nebraska (*n* = 37) using standardized drag sampling during fall 2024 and spring 2025. Dragging was conducted for approximately 1–4 h per site (mean ~2 h), and drag cloths were examined for ticks at 25 m intervals. All collected ticks were preserved in 70% ethanol and identified to species and life stage in the laboratory. Additionally, deer checks were conducted during fall 2024 at designated check stations to assess host‐associated tick presence. All ticks collected from a single deer were placed into a vial containing 70% ethanol, and tick species, sex, life stage and total counts were recorded per deer. All infested deer were killed in Dodge, Washington and Douglas counties. Host‐seeking specimens from Iowa (*n* = 19) were collected as described for Nebraska using active tick drags in April or June of 2023. Live ticks were placed in vials and stored at −80°C prior to shipping to UNMC. Host‐seeking ticks were collected in South Dakota (*n* = 5) by flagging vegetation. Following species identification, ticks were cut in half with a scalpel and frozen at −20°C or −80°C prior to shipping to UNMC. For specimens from Kansas (*n* = 5), field collection and preservation methods were previously described (Ng'eno et al. 2024; McClung et al. 2023). In addition to field‐collected specimens, live nymphal ticks (*n* = 3) were obtained through BEI Resources from a laboratory strain originally established using ticks collected from Rhode Island. Nymphs were killed via freezing at −80°C prior to homogenization and gDNA purification.

**FIGURE 1:**
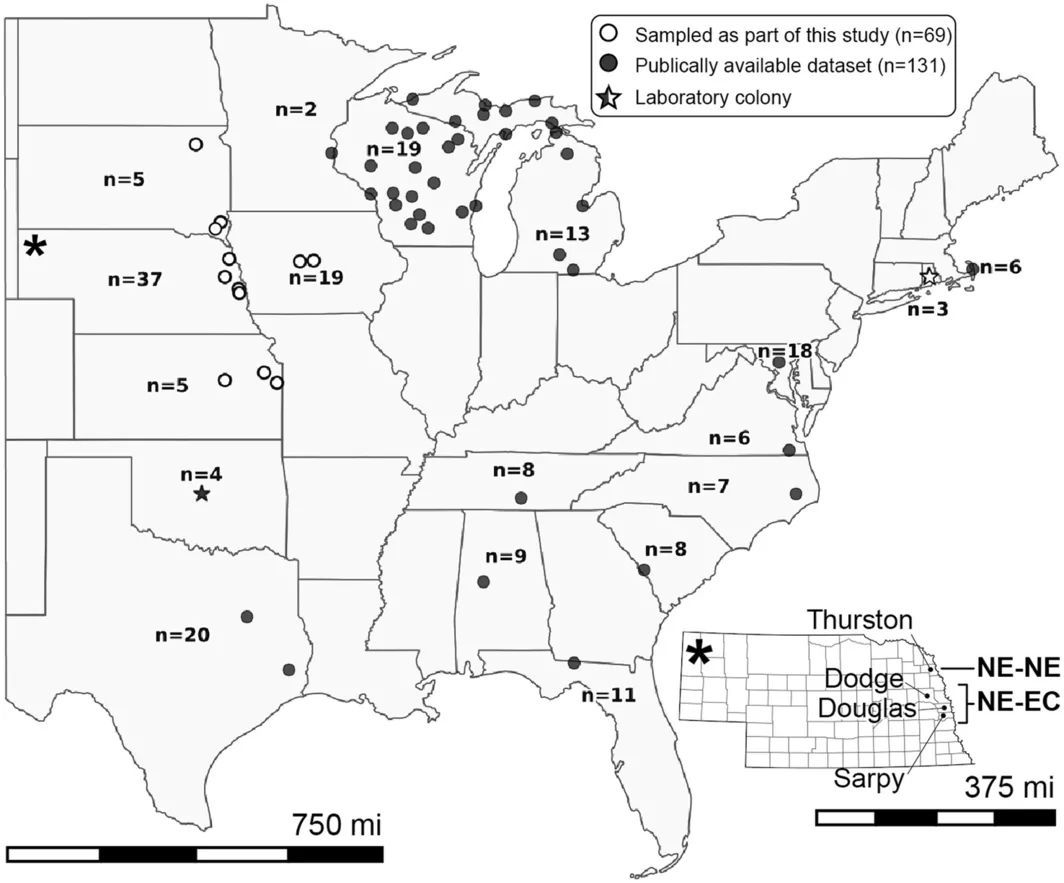
Sampling localities for individuals of *Ixodes scapularis* included in population genomic analyses. Circles indicate field collection sites; stars indicate laboratory colonies. Open shapes indicate localities that were newly‐sampled as part of this study; closed shapes indicate localities for publicly‐available datasets that were downloaded for inclusion. Values of n indicate the total number of individuals of *I. scapularis* included from each state. Detailed map at right, denoted by asterisk (*) shows county‐level designations for each sampling site in Nebraska, labelled by region (northeastern Nebraska, NE–NE; or east central Nebraska, NE–EC).

### 2.2 Sample Processing

Individual ticks underwent surface sterilization using sequential washes of 3% H_2_O_2_ (1 min), 70% ethanol (two 30‐s washes) and 1× PBS (2 min) with vortexing between each step. Sterilized ticks were homogenized in 100 μL phosphate buffered saline (PBS; 1×; pH 7.4; Thermo Fisher Scientific) in Safe‐Lock Tubes (Eppendorf) using a TissueLyser II tissue disruptor (QIAGEN) with stainless steel beads at 25 Hz for 5, 10 or 30 min (see Table S1). Total genomic DNA was purified from homogenized tissue with two approaches. The first approach used the DNeasy Blood & Tissue Kit (Qiagen) following the manufacturer's protocol with overnight proteinase K digestion at 56°C and elution in 50 μL AE Buffer. The second approach used the Zymo Quick DNA HMW MagBead Kit (Zymo Research) and the modified purification protocol previously described (Herzog et al. 2024). Briefly, for ticks extracted using the Zymo Kit, homogenization was done in 95 μL Zymo Biofluid & Solid Tissue Buffer rather than PBS. Following homogenization, samples were digested overnight with proteinase K digestion at 56°C with occasional mixing. Following the addition of MagBinding Beads, samples were placed on a benchtop rotator for 1.5 HR at room temperature. Elutions were performed at 37°C overnight. Purified gDNA from both approaches were quantified using a Qubit double‐stranded high sensitivity DNA assay (dsDNA HS; Invitrogen). For the specimens from Kansas (*n* = 5; see Table SS1), gDNA was previously extracted as described by McClung et al. (2023). Prior to library preparation, these extractions were bead‐cleaned using KAPA Pure Beads (Roche) in a 1:1 ratio, eluted in nuclease‐free water and quantified using Qubit dsDNA HS assay.

### 2.3 Next‐Generation Sequencing Library Preparation

For each specimen, 15–40 ng of gDNA was used as input to library preparation (see Table S1). Individual WGS libraries were prepared using an Illumina DNA Prep Kit (Illumina) following the manufacturer's protocol with 6 or 8 cycles of unique dual‐indexing PCR, depending on gDNA input (see Table S1). Purified indexed libraries were quantified via Qubit dsDNA HS assay and pooled for equal concentration. Pools then underwent an additional round of double‐sided bead purification following the ratios and incubation times given in the Illumina DNA Prep Kit protocol. Bead‐purified pools were quantified via Qubit dsDNA HS assay and the KAPA Library Quantification Kit for Illumina Platforms (KAPA Biosystems). Insert length was assessed with High Sensitivity D1000 DNA ScreenTape analysis (Agilent) on an Agilent 2200 TapeStation system.

### 2.4 Next‐Generation Sequencing

Pooled libraries were submitted to the University of Nebraska Medical Center Genomics Core Facility (UNMC GCF) for 2 × 150 paired‐end Illumina sequencing in two rounds. For the first round, the bead‐purified pool (*n* = 61 libraries) was sequenced on an Illumina NovaSeq 6000 S2 flow cell. For the second round, the bead‐purified pool (*n* = 8 libraries) was sequenced on an Illumina NovaSeq 6000 S1 flow cell. For each round, 10× depth of coverage (DOC) across the *I. scapularis* genome was targeted per individual. Data were demultiplexed by the UNMC GCF and raw reads from the same libraries sequenced across multiple lanes were concatenated prior to analysis.

### 2.5 Integration of Previously Generated Data

To establish a comprehensive population framework and contextualize our newly generated data, we integrated publicly available WGS Illumina data from the National Center for Biotechnology Information Sequence Read Archive (NCBI SRA). For *I. scapularis* , 131 datasets were selected, providing coverage across 13 states representing the established northern and southern clades (see Figure 1, Table S1) (Schoville et al. 2024; Guzman‐Valencia et al. 2026; Tietjen et al. 2025). Raw reads were downloaded using fasterq‐dump through the NCBI SRA Toolkit v2.11 (National Center for Biotechnology Information n.d.).

### 2.6 Data Quality Control and Reference Alignment

Illumina sequence datasets generated herein (*n* = 69) and downloaded from the NCBI SRA (*n* = 131) were processed using the same bioinformatic pipeline (see Figure S1). Raw reads were quality‐filtered using fastp v0.23 (Chen et al. 2018) to trim adaptor sequences and remove low‐quality reads and reads shorter than 50 base pairs (bp). FastQC v0.12 (Bioinformatics 2011) was then used to confirm successful trimming and filtration. Repeat regions in the *I. scapularis* reference genome (ASM1692078v2) (De et al. 2023) were masked using RepeatMasker v4.0 (*RepeatMasker* n.d.) and then quality‐controlled reads were aligned to the masked reference using BWA‐MEM v0.7 (Li and Durbin 2009) with default parameters. To minimize potential biases arising from uneven sequencing depth across samples, the eight libraries sequenced on an S1 flow cell that achieved substantially higher coverage (~30×) than the target depth were downsampled to match the coverage distribution of the remaining samples (~8×). Downsampling fractions were calculated for each sample based on original read counts, targeting approximately 135 million reads per sample. Downsampling was performed using samtools v1.20 with the ‐s flag, which randomly subsamples reads using a fixed seed (seed = 42) to ensure reproducibility. Resulting sequence alignment map (SAM) files were converted to BAM format, coordinate‐sorted and indexed using samtools v1.19 (Li et al. 2009). Read groups were then added using Picard v3.0 (Picard n.d.) AddOrReplaceReadGroups and PCR duplicates were removed using Picard MarkDuplicates with the ‘‐‐OPTICAL_DUPLICATE_PIXEL_DISTANCE 2500’ flag appropriate for patterned flow cells.

### 2.7 Variant Detection and Single Nucleotide Polymorphism Isolation and Filtration

Variant calling was performed using the Genome Analysis Toolkit (GATK) v4.6 (McKenna et al. 2010) following the best practices workflow for germline short variant discovery (DePristo et al. 2011; Van der Auwera et al. 2013). First, HaplotypeCaller in GVCF mode with diploid genome modelling was used to generate gVCFs for individual samples. This process was run across scattered genomic intervals using SplitIntervals with a scatter count of 20. The resulting gVCFs were consolidated into a GenomicsDB workspace using GenomicsDBImport. Joint genotyping across all samples was performed using GenotypeGVCFs. Individual VCFs from all samples were then gathered into a final jointly‐called variant dataset using GATK GatherVcfs. Indels were removed using SelectVariants to retain only single nucleotide polymorphisms (SNPs) for downstream analysis. Baseline quality filters were applied following GATK best practices, flagging variants that failed any of the following criteria: QD < 2.0, QUAL < 30.0, SOR > 3.0, FS > 60.0, MQ < 40.0, MQRankSum < −12.5 or ReadPosRankSum < −8.0. Flagged variants were then removed using SelectVariants. Additional site‐level filtration was performed using VCFtools v0.1.16 (Danecek et al. 2011) to retain only variants with MAF > 0.05, MAC 2, biallelism, site‐level missingness > 5%. Filtered datasets were then used for downstream analysis with standard population genomic software, some of which required additional filtering (see Figure SS1).

### 2.8 Population Summary Statistics

Summary statistics and corrected F_ST_ values were generated using the ‘populations’ module in STACKS v2.68 (Catchen et al. 2013; Rochette et al. 2019). The module was run using a population grouping scheme where each state was considered its own population, with Nebraska specimens designated as either a northeastern population (NE–NE) or an east central population (NE‐EC) (*n* = 19 populations total; see Table S1). At least 80% of specimens within a population were required to have a genotype call in order to process a locus for that population, and at least 13 of 19 populations were required to be represented at a locus for inclusion. Kernel smoothing was enabled for *F*‐statistic calculation and *F*_ST_ correction was implemented with a *p*‐value cutoff of 0.05.

### 2.9 Principal Components‐Based Analyses

Principal components analysis (PCA) was conducted using PLINK v2.0a1 (Chang et al. 2015). For PCA, the filtered SNP dataset was converted to binary format and linkage disequilibrium (LD) pruning was performed using a sliding window approach (window size of 50 kb, window step size of 10 bp and *r* ^2^ threshold of 0.1). The resulting PCA plot was visualized using matplotlib v3.10.0 (Hunter 2007) and seaborn v0.13.2 (Waskom 2021). Discriminant analysis of principal components (DAPC) was performed using the R package adegenet v2.1.11 (Jombart 2008; Jombart and Ahmed 2011) in R v4.5.1 (R Core Team 2023). For DAPC, the most likely number of populations was determined using both Bayesian information criterion (BIC) and Akaike information criterion (AIC) and the number of retained principal components was determined through a‐score optimization.

### 2.10 Admixture Analyses

Admixture analysis was performed using fastSTRUCTURE v1.0 (Raj et al. 2014) and ADMIXTURE v1.3 (Alexander et al. 2009). To ensure that fastSTRUCTURE could be reliably used in place of STRUCTURE v2.3 (Pritchard et al. 2000; Falush et al. 2003), a preliminary analysis was performed on a reduced dataset to compare outputs from both programmes. For both reduced and full datasets, VCFs were first filtered for LD as described above using PLINK2, then converted to STRUCTURE format using PGDSpider v2.1.1.5 (Lischer and Excoffier 2012) with a custom SPID configuration file specifying diploid genotype handling, SNP‐only data export and exclusion of monomorphic SNPs. Missing data were preserved and coded as ‘‐9’. No population definition file was used during conversion to allow for *de novo* inference of admixture. Following conversion, the STRUCTURE format file was manually edited to remove the first row containing SNP identifiers as these labels can interfere with proper file parsing. For STRUCTURE using the reduced dataset, *k*‐values 1–5 were tested with 10 independent runs completed for each *k*‐value, with 250,000 generations for each run, with the first 50,000 generations discarded as burn‐in. The most likely *k*‐value was determined using likelihood scores and the Evanno Δ*K* method (Evanno et al. 2005) implemented in STRUCTURE HARVESTER v.0.6.93 (Earl and vonHoldt 2012). For fastSTRUCTURE using the reduced and full dataset, *k*‐values 1–10 were tested using the simple prior with default convergence criterion and the full likelihood model. The optimal *k*‐value was determined using two complementary approaches implemented in the chooseK.py script: (1) the *k*‐value that maximized the variational lower bound (marginal likelihood) and (2) the modal *k*‐value across runs based on the number of model components required to explain structure in *N* − 1 individuals from the admixture proportions (Q matrices). Population membership coefficients were visualized using the district.py script included with fastSTRUCTURE, with samples grouped and ordered by state. For ADMIXTURE, only the full LD‐pruned PLINK2 fileset (.bed/.bim/.fam) was used as input; however, because ADMIXTURE requires integer chromosome codes, scaffold names were converted to sequential integers prior to analysis using a custom mapping file. ADMIXTURE was run for *k* = 1–6 using 8 threads, with 5‐fold cross‐validation (‐‐cv) enabled to assess model fit. The optimal *k*‐value was determined by identifying the *k* with the lowest cross‐validation error. Ancestry proportion estimates (Q matrices) were visualized with samples grouped by state for comparison with fastSTRUCTURE results.

### 2.11 Isolation by Distance Visualization

To visualize isolation by distance (IBD), the geographic distance between populations was calculated using the Haversine formula and plotted against pairwise corrected *F*_ST_ values generated via STACKS (see above). For populations represented by two or three sampling localities (Iowa, Kansas, east central Nebraska [NE‐EC], South Dakota, Texas), the geographic coordinates of the midway point among sampling localities were used for distance calculations and for populations represented by more than three sampling localities (Michigan, Wisconsin) the geographic coordinates for the centroid of the state were used. Each state‐based population was characterized as either ‘majority northern descent’ or ‘majority southern descent’ based on results from principal component‐ and admixture‐based analyses, with the NE‐EC population defined as evenly split between the two. Pairwise comparisons representing majority northern descent versus majority southern descent populations were highlighted separately from all other comparisons. Populations represented by laboratory colonies (Rhode Island–BEI, Oklahoma–OSU) were excluded from IBD estimations.

### 2.12 Determination of *Borrelia burgdorferi* Infection Rates From Whole Genome Sequencing Data

To identify reads from associated *B. burgdorferi* bacteria, NGS were aligned to a multireference file that contained both the *I. scapularis* reference genome (ASM1692078v2) and the *B. burgdorferi* reference genome (GCA_000181575.2, Bol26). Reads were aligned using BWA‐MEM v0.7, and regions corresponding to *B. burgdorferi* were extracted to .bam files and filtered with samtools view for alignment quality with the following flags: ‐q 20 ‐e ‘(qlen‐sclen) > 75’. The number of reads aligning to each *B. burgdorferi* genome/plasmid sequence and the total number of reads aligning to *B. burgdorferi* overall were calculated for each sample using samtools flagstat. Identifying multiple reads across multiple genomic and/or plasmid contigs was used as an indication for infection with *B. burgdorferi* .

## 3 Results

### 3.1 DNA Extraction and Library Preparation

In total, we generated 69 novel WGS datasets from individual specimens of *I. scapularis* , collectively from Nebraska (*n* = 37), Iowa (*n* = 19), Kansas (*n* = 5), South Dakota (*n* = 5) and the BEI Resources Rhode Island‐derived laboratory strain (*n* = 3) (Figure 1, Table S1). The specimens from Nebraska represent both the northeastern (Thurston County, *n* = 11; NE–NE) and east central (Dodge, Douglas and Sarpy Counties; *n* = 26; NE‐EC) regions of the state. Specimens from Nebraska, Iowa, Kansas and South Dakota represent the first complete WGS data for *I. scapularis* generated from each of these states. DNA extraction efficiency differed between methodological approaches and life‐cycle stages. Median gDNA yield from adult ticks extracted with the Qiagen DNeasy Blood & Tissue kit was 140.0 ng, with substantial variation across samples. In contrast, nymphal ticks and sets of eight legs from single adult ticks extracted with the Zymo Quick‐DNA HMW MagBead Kit yielded medians of 106.0 and 70.8 ng of gDNA, respectively, with lower among‐sample variance from substantially smaller input tissue mass (Figure S2).

### 3.2 Next‐Generation Sequencing, Incorporation of Publicly‐Available Data and Variant Calling

Sequencing of the 69 WGS libraries yielded 12.6 billion read pairs total, with 102.1–637.7 million read pairs generated per specimen (mean 182 million read pairs). Quality filtration with fastp resulted in a high proportion of data retention (95.8%–97.5%; 96.5% ± 0.4%), with low duplication rates (5.0%–8.2%; 6.2% ± 0.7%). Following alignment to the repeat‐masked *I. scapularis* reference genome, mapping rates were consistently high (90.7%–99.0%; 98.5% ± 1.0%). Mean depth of coverage was 10.8× ± 7.3× (6.1–37.7×; median 8.4×) with 55.8% ± 9.4% of the genome covered at > 5× and 29.8% ± 16.0% at > 10×. To establish a comprehensive population framework, we integrated 131 publicly available WGS datasets from the NCBI SRA, representing 13 states across the established range of *I. scapularis* (Figure 1). These datasets yielded 10.6 billion raw read pairs total, with 32.4–701.6 million read pairs per specimen (mean 80.7 million read pairs). Data retention after quality filtration was high (96.0%–99.9%; 99.3% ± 0.8%) though duplication rates were more variable as compared to the datasets generated as part of this study (5.8–23.7; 10.9% ± 3.5%). Mapping rates were comparable to those of the newly‐generated datasets (96.1%–99.3%; 98.6% ± 0.6%). Mean depth of coverage ranged from 2.0 to 10.5× (4.0× ± 1.4×; median 3.9×), with 21.8% ± 10.7% of the genome covered at > 5× and 6.2% ± 5.7% of the genome covered at > 10×. Joint variant calling across all 200 specimens resulted in 527,287,655 SNPs. Following stringent quality filtration using GATK and VCFtools, 333,778 high‐quality SNPs were retained. Linkage disequilibrium pruning with PLINK2 further reduced the dataset to 123,186 SNPs for PCA‐ and admixture‐based analyses (Table 1).

**TABLE 1:** Number of single nucleotide polymorphisms (SNPs) retained after each step of SNP filtration.

Unfiltered	GATK baseline quality filtration	MAF > 0.05, MAC 2, biallelism, site‐level missingness > 5%	Linkage disequlibrium
527,287,655	423,746,161	333,778	123,186

*Note:* For PCA, DAPC, fastSTRUCTURE and ADMIXTURE analysis, the fully‐filtered dataset through linkage disequilibrium filtration was used (*n* = 123,186 SNPs); for generation of population‐level summary statistics using the STACKS populations module, no linkage disequilibrium filter was applied (*n* = 333,778 SNPs).

### 3.3 Population‐Level Summary Statistics

Values for proportion of polymorphic sites, observed heterozygosity (H_O_), expected heterozygosity (H_E_), inbreeding coefficients (F_IS_) and nucleotide diversity (*π*) considering variant and fixed sites for all subpopulations are presented in Table 2. As expected, a greater proportion of polymorphic sites was generally identified in larger populations. Exceptions include Texas and Florida, which are each relatively large subpopulations (> 10 specimens per site analysed on average) but had comparatively low proportions of polymorphic sites (< 50%). Observed heterozygosity and nucleotide diversity were generally lower for subpopulations in southern geographic regions (H_O_: 0.047–0.16, *π*: 0.08–0.155) as compared to those in northern regions (H_O_: 0.087–0.215, *π*: 0.134–0.199), suggesting greater standing genetic diversity in northern regions. Inbreeding coefficients were near 0 for all populations, indicating no significant signature of inbreeding. This was true even for the two populations representing laboratory strains (Rhode Island–BEI, Oklahoma–OSU). For populations from the Great Plains, relatively high values for observed heterozygosity and nucleotide diversity were inferred (H_O_: 0.19–0.217, *π*: 0.184–0.2), consistent with expectations for admixed populations.

**TABLE 2:** Population‐level summary statistics generated from *Ixodes scapularis* single nucleotide polymorphism (SNP) datasets (*n* = 333,778 SNPs).

	Population	Avg no. individ./site	No. sites	% polymorphic sites	H_O_	H_E_	*F*_IS_	*π*
North	RI (BEI)	3.0	325,246	34.48	0.207	0.138	−0.071	0.165
	MN	2.0	326,752	35.97	0.215	0.149	−0.024	0.199
	WI	18.2	329,320	69.52	0.139	0.161	0.135	0.166
	MI	12.6	330,184	70.83	0.152	0.165	0.101	0.172
	MA	5.7	311,750	45.19	0.119	0.134	0.077	0.147
	MD	16.5	298,131	63.52	0.087	0.130	0.222	0.134
	IA	18.8	333,008	84.87	0.213	0.191	−0.018	0.196
Great Plains	SD	4.9	332,787	61.39	0.216	0.180	−0.028	0.200
	NE–NE	10.9	332,970	74.36	0.217	0.189	−0.032	0.198
	NE–EC	25.6	333,046	92.25	0.190	0.181	0.015	0.184
	KS	4.9	332,663	47.05	0.160	0.140	−0.008	0.155
South	TN	7.7	315,883	50.11	0.107	0.125	0.088	0.133
	VA	5.8	326,334	37.92	0.106	0.108	0.038	0.118
	NC	6.7	318,723	36.66	0.088	0.099	0.060	0.107
	SC	7.7	310,557	38.73	0.087	0.102	0.075	0.109
	AL	8.7	309,056	41.10	0.094	0.106	0.065	0.112
	FL	10.5	327,404	43.30	0.090	0.104	0.079	0.110
	TX	18.5	327,667	50.38	0.072	0.099	0.150	0.102
	OK (OSU)	4.0	195,523	20.49	0.047	0.070	0.069	0.080

*Note:* Individuals were assigned to populations by state of origin, with individuals from Nebraska designated as either a northeastern region (NE–NE; Thurston county) or east central region (NE–EC; Dodge, Douglas or Sarpy counties) population. Individuals from laboratory colonies (Rhode Island, BEI; and Oklahoma, OSU) are labelled with their associated laboratory strain acronyms.

### 3.4 Principal Components‐Based Analyses

PCA revealed clear population structure among the 200 individual *I. scapularis* . The classic horseshoe shape of the plot indicates isolation by distance among these populations. The first two principal components explained 29.2% and 6.6% of the total variance in the dataset, respectively and differentiated samples into two primary clusters corresponding to northern and southern geographic origins (Figure 2). Specimens from northern states (Massachusetts, Rhode Island [BEI], Maryland, Michigan, Wisconsin and Minnesota) grouped together, as did specimens from southern states (Texas, Oklahoma [OSU], Alabama, Virginia, North Carolina, South Carolina and Florida). Specimens from Tennessee showed intermediate grouping between the two clusters, though with closer identity to the southern cluster. Newly‐sequenced specimens (depicted with triangles in Figure 2) showed varying placement. Among specimens from Nebraska, geographic sub‐structuring was evident. Ticks from northeastern Nebraska (Thurston County; NE–NE) grouped with the northern cluster, while ticks from east central Nebraska (Dodge, Douglas and Sarpy Counties; NE‐EC) showed intermediate positioning between the two clusters. Specimens from Kansas were also intermediately positioned, while those from South Dakota and Iowa clustered largely with the northern clade.

**FIGURE 2:**
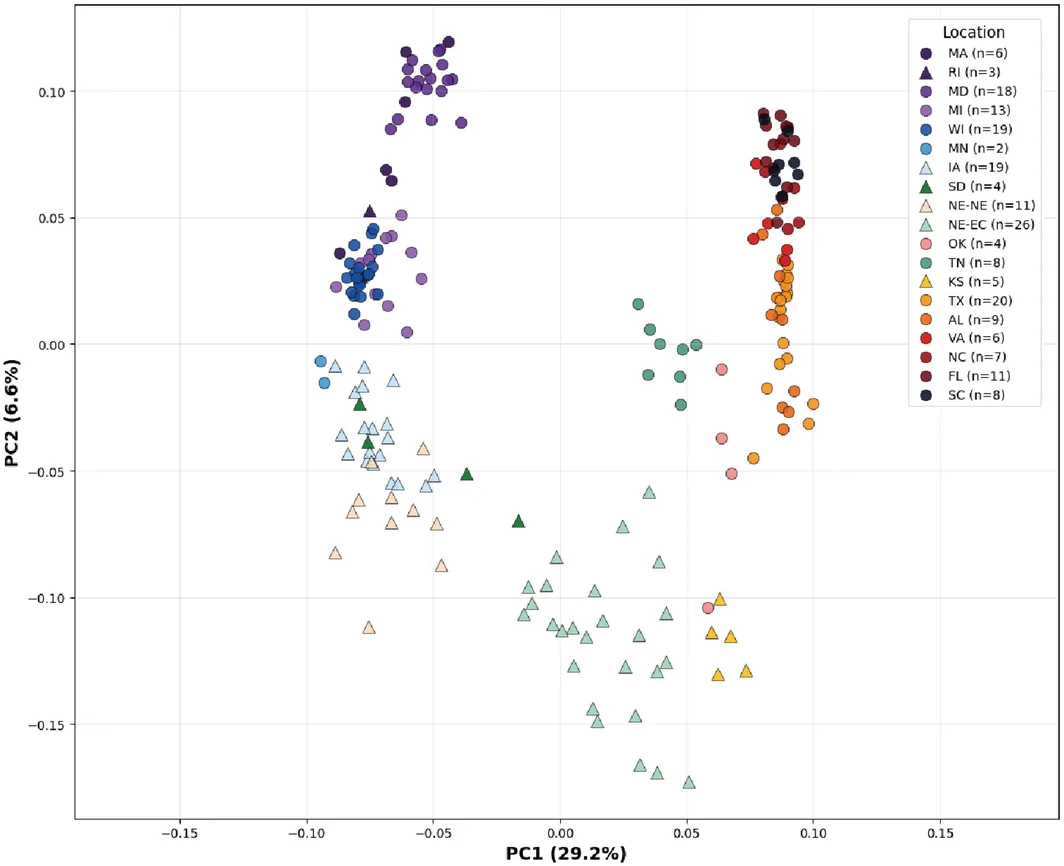
Principal components analysis (PCA) generated with PLINK2 from single nucleotide polymorphism (SNP) datasets for *Ixodes scapularis* based on 123,186 SNPs. Each shape represents a single individual of *I. scapularis* . Shapes are coloured by the state of collection for each individual, with Rhode Island and Oklahoma represented by individuals obtained from laboratory colonies rather than field collections. Individuals collected from Nebraska are designated as either from the northeastern region (NE–NE; Thurston county) or east central region (NE–EC; Dodge, Douglas or Sarpy counties). Triangles indicate individuals sequenced as part of this study; circles indicate individuals for which previously‐deposited data were downloaded for inclusion.

For DAPC, BIC optimization supported two as the most likely number of genetically distinct clusters, while AIC optimization showed a slight preference for four clusters (Figure S3). Results from DAPC for both a two‐cluster (Figure 3A) and four‐cluster (Figure 3B) analysis are presented here. For the two‐cluster analysis, the resulting discriminant function clearly separated specimens from northern versus southern geographic regions with minimal overlap, with most specimens (*n* = 191 of 200; 95.5%) assigned to one cluster with > 96% identity. These cluster assignments largely aligned with the results from PCA, with exceptions for specimens from Tennessee and Nebraska. Specimens from Tennessee grouped confidently with the southern cluster (99.9%–100% cluster identity) while specimens from Nebraska showed variation in cluster assignment. All 11 specimens from northeastern Nebraska (Thurston County; NE–NE) and three specimens from east central Nebraska (Dodge County; NE‐EC) grouped with the northern cluster (96.7%–100% cluster identity) and 14 specimens from east central Nebraska (Dodge, Douglas or Sarpy Counties) grouped with the southern cluster (96.4%–100% identity). The remaining specimens from east central Nebraska (*n* = 9) showed mixed cluster assignment, ranging from 90.7% identity with the majority‐southern cluster to 84.7% identity with the majority‐northern cluster (see Figure 3A). In the four‐cluster analysis, clusters also aligned with geographic boundaries. Cluster 2 consisted of majority‐Northeastern specimens (Rhode Island [BEI], Maryland and Massachusetts), Cluster 1 consisted of majority‐Upper Midwest and northern Great Plains specimens (Michigan, Minnesota, Wisconsin, Iowa, South Dakota and northeastern Nebraska [NE–NE]) and Cluster 4 consisted of majority‐southern specimens (Virginia, North Carolina, South Carolina, Alabama, Florida, Texas and Oklahoma [OSU]) with high cluster assignment identity (> 89.6%–100%) for nearly all specimens. The exceptions were a specimen from Michigan, which had 74.5% and 25.5% assignment to clusters 2 and 1, respectively and a specimen from the OSU laboratory colony, which had 76.2% and 23.8% assignment to clusters 4 and 3, respectively. Cluster 3 is composed of specimens from Tennessee, South Dakota, east central Nebraska (NE‐EC) and Kansas, and three individuals from the OSU laboratory colony, with each individual demonstrating 62.6%–100% cluster assignment identity. This result aligns with the intermediary positioning of these specimens observed in the PCA (see Figure 2).

**FIGURE 3:**
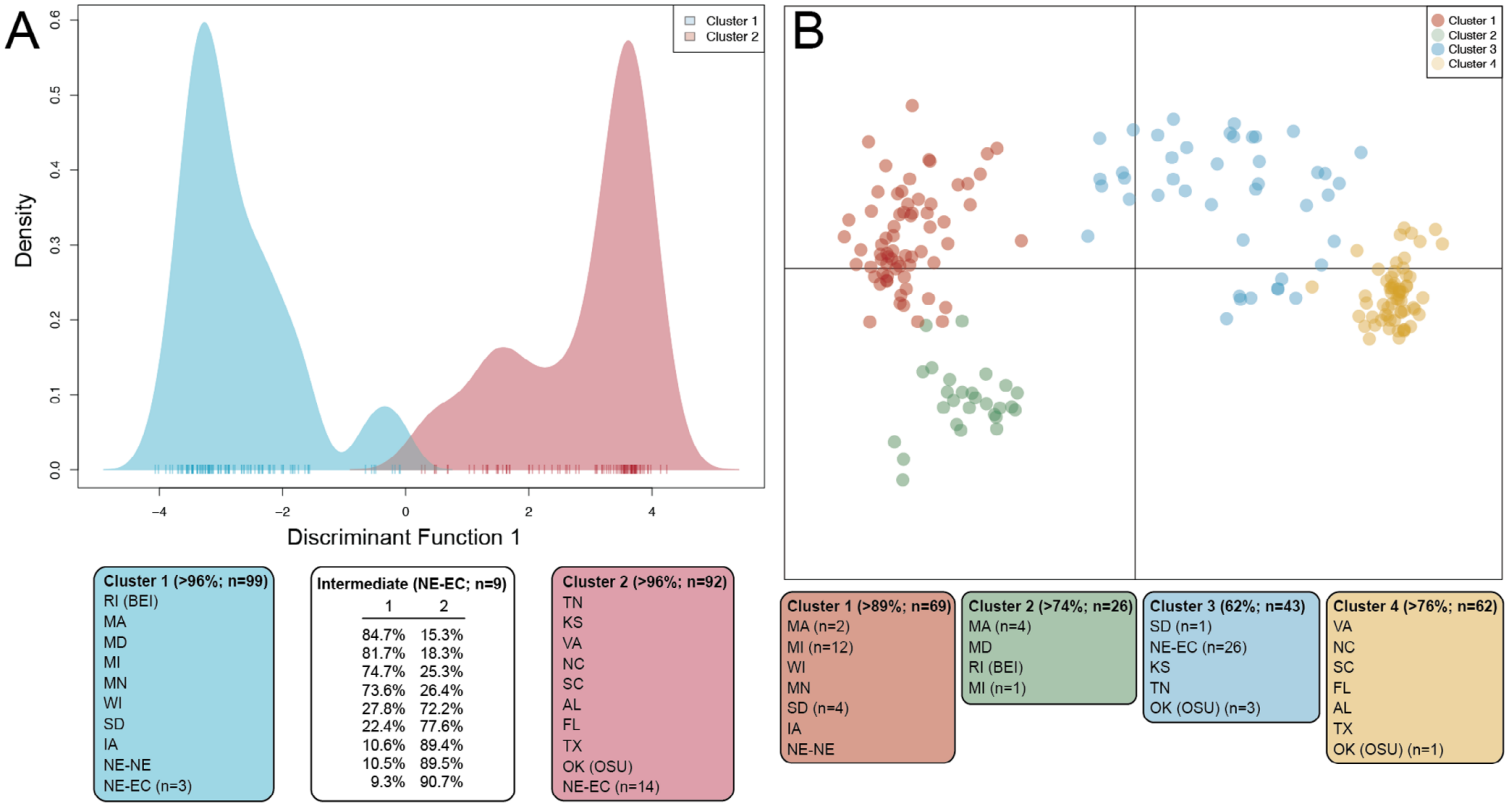
Discriminant analysis of principal components (DAPC) plots from single nucleotide polymorphism (SNP) datasets for *Ixodes scapularis* based on 123,186 SNPs. (A) Bayesian information criterion (BIC)‐preferred cluster number of two; colour‐coded lists below plot show individuals assigned to each cluster above a specified threshold of identity; values of n provided for a state indicate that individuals from that population were assigned across multiple clusters; table at center shows individuals from east central Nebraska (NE–EC) of intermediate cluster identity with their cluster assignment proportions. (B) Akaike information criterion (AIC)‐preferred cluster number of four; colour‐coded lists below plot show individuals assigned to each cluster above a specified threshold of identity; values of n provided for a state indicate that individuals from that population were assigned across multiple clusters.

### 3.5 Admixture Analyses

Analysis of the reduced dataset to compare STRUCTURE and fastSTRUCTURE outputs produced nearly identical results (see Figure SS4), indicating fastSTRUCTURE as a reliable alternative to STRUCTURE for the full dataset. Proportions of ancestry assignment for each individual in the full dataset were largely concordant between fastSTRUCTURE and ADMIXTURE, and aligned with geographic region of origin (Figure 4). For fastSTRUCTURE, log‐marginal likelihood lower bound (LLBO) and optimal components estimation (KΦ*) supported *k* = 2 (Figure S5). For ADMIXTURE, cross‐validation (CV) errors also supported *k* = 2 (Figure S5). At *k* = 2, specimens from the Northeast (Massachusetts, Maryland, Rhode Island [BEI]) and the Upper Midwest (Michigan, Wisconsin, Minnesota) shared high proportions of ancestry assignment to a common ancestral population (Figure 3; majority blue or purple bars). Similarly, specimens from southern states (Texas, Oklahoma [OSU], Alabama, Virginia, North Carolina, South Carolina and Florida) showed high proportions of ancestry assignment to the second inferred ancestral population (Figure 3; majority red or green bars). Specimens from Iowa and South Dakota were assigned a majority (> 50%) proportion of ancestry to the same ancestral population as Northeast and Upper Midwest specimens, while specimens from Kansas and Tennessee had majority ancestry assignments that aligned with those of specimens from southern states. Specimens from Nebraska displayed clear evidence of admixture, with ancestry assignments varying substantially within the state. Specimens from northeastern Nebraska (Thurston County; NE–NE) had ancestry assignments similar to those of specimens from northern regions, while specimens from east central Nebraska (Dodge, Douglas and Sarpy Counties; NE‐EC) displayed mixed ancestry, with assignment proportions near 50% for each individual.

**FIGURE 4:**
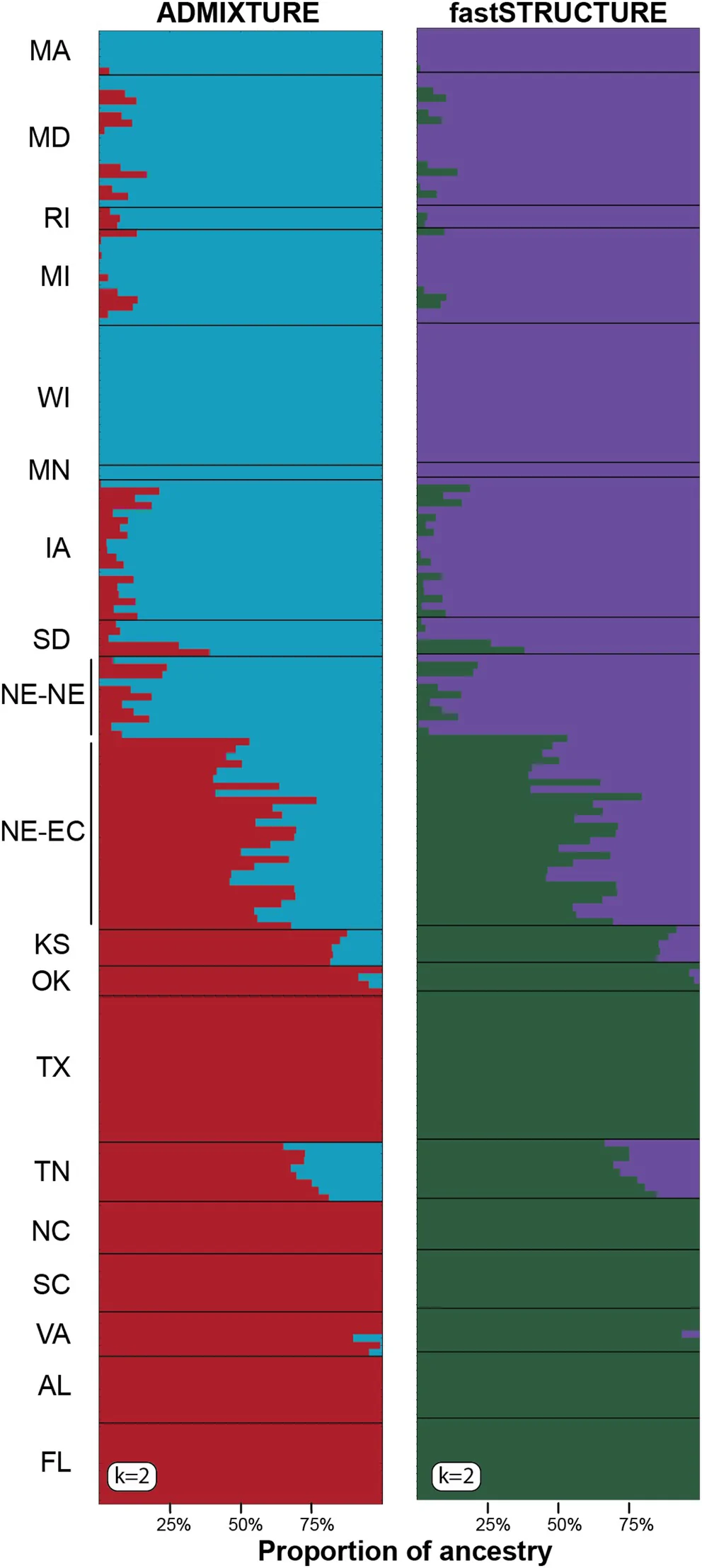
Admixture plots from single nucleotide polymorphism (SNP) datasets for *Ixodes scapularis* based on 123,186 SNPs generated with ADMIXTURE (left) and fastSTRUCTURE (right). For both analyses, the statistically preferred value of *k* = 2 for ancestry assignment is shown. Abbreviations for state of origin for individual ticks are given at left with Nebraska designated as either a northeastern region (NE–NE) or east central region (NE–EC) population.

### 3.6 *F*_ST_ Estimation and Visualization of Isolation by Distance

Corrected *F*_ST_ values estimated for each pair of populations are summarized in Table 3. On average, the greatest *F*_ST_ values (0.024–0.111; mean 0.053) were estimated between populations with majority‐northern ancestry estimation (Rhode Island [BEI], Minnesota, Wisconsin, Michigan, Massachusetts, Maryland, South Dakota, Iowa and northeastern Nebraska) and majority‐southern ancestry estimation (Kansas, Tennessee, Virginia, North Carolina, South Carolina, Alabama, Florida and Texas). Values among majority‐northern ancestry populations (0.004–0.039; mean 0.015) and among majority‐southern ancestry populations (0.007–0.035; mean 0.022) were comparatively lower and similar between the two groups, with a higher average *F*_ST_ estimated among majority‐southern ancestry populations. As expected, values were slightly elevated for all comparisons to both the BEI (0.023–0.111; mean 0.057) and OSU (0.006–0.058; mean 0.032) laboratory colonies. Relatively low *F*_ST_ values were estimated for all comparisons against the east‐central Nebraska population (0.005–0.03; mean 0.016), consistent with expectations for genomic admixture in this region. Plotting estimated pairwise *F*_ST_ values against physical distance between populations to visualize IBD clearly shows elevated *F*_ST_ values for populations in northern versus southern regions, though *R* ^2^ values (0.1315 and 0.0179, respectively) do not indicate a strong correlation between distance and *F*_ST_ value for either set of comparisons (Figure 5).

**TABLE 3:** Corrected pairwise *F*_ST_ values from *Ixodes scapularis* single nucleotide polymorphism (SNP) datasets (*n* = 333,778 SNPs).

	MN	WI	MI	MA	MD	SD	IA	NE–NE	NE–EC	TN	KS	VA	NC	SC	AL	FL	TX	OK (OSU)
RI (BEI)	0.030	0.037	0.033	0.036	0.039	0.025	0.023	0.030	0.027	0.061	0.060	0.082	0.087	0.088	0.087	0.111	0.107	0.058
MN		0.006	0.008	0.008	0.021	0.004	0.005	0.006	0.012	0.057	0.026	0.035	0.077	0.086	0.085	0.082	0.080	0.046
WI			0.005	0.011	0.019	0.013	0.006	0.011	0.030	0.044	0.052	0.050	0.052	0.056	0.061	0.065	0.070	0.046
MI				0.008	0.014	0.005	0.004	0.008	0.022	0.035	0.035	0.041	0.046	0.048	0.052	0.055	0.064	0.037
MA					0.011	0.007	0.006	0.011	0.015	0.037	0.036	0.051	0.052	0.053	0.055	0.063	0.067	0.046
MD						0.016	0.014	0.024	0.024	0.033	0.042	0.041	0.041	0.044	0.050	0.050	0.052	0.040
SD							0.004	0.006	0.010	0.029	0.024	0.033	0.034	0.045	0.051	0.052	0.070	0.027
IA								0.007	0.022	0.028	0.028	0.033	0.036	0.040	0.046	0.051	0.061	0.026
NE–NE									0.020	0.036	0.030	0.040	0.042	0.047	0.052	0.060	0.073	0.029
NE–EC										0.011	0.005	0.011	0.012	0.014	0.013	0.020	0.022	0.006
TN											0.015	0.021	0.026	0.027	0.028	0.031	0.031	0.026
KS												0.016	0.016	0.023	0.026	0.030	0.028	0.011
VA													0.007	0.010	0.019	0.018	0.019	0.033
NC														0.012	0.019	0.016	0.016	0.033
SC															0.020	0.009	0.018	0.031
AL																0.026	0.019	0.028
FL																	0.024	0.035
TX																		0.027

*Note:* Individuals were assigned to populations by state of origin, with individuals from Nebraska designated as either a northeastern region (NE–NE; Thurston county) or east central region (NE–EC; Dodge, Douglas or Sarpy counties) population. Individuals from laboratory colonies (Rhode Island, BEI; and Oklahoma, OSU) are labelled with their associated laboratory strain acronyms. Greater values are coloured a deeper shade of orange.

**FIGURE 5:**
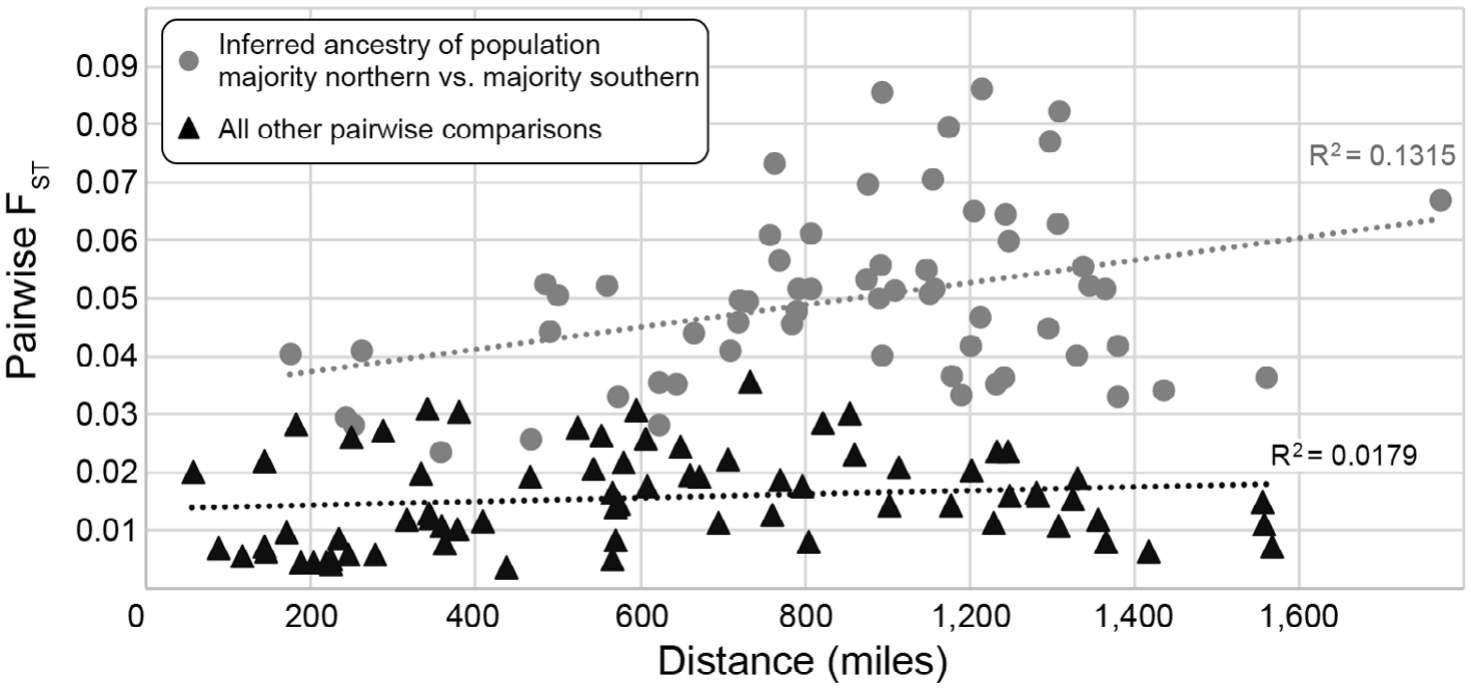
Isolation by distance plot for *Ixodes scapularis* . Pairwise comparisons are made between all populations included in analyses except for ticks from laboratory‐reared colonies (Rhode Island; BEI and Oklahoma; OSU). Comparisons between populations with inferred ancestries that are majority northern vs. majority southern according to principal components‐ and admixture‐based analyses are shown in grey circles; all other comparisons are shown as black triangles. Trendlines for both groups are presented as grey or black dotted lines labelled with *R*‐squared values.

### 3.7 *Borrelia burgdorferi* Infection Rates Inferred From Sequencing Data

Analysis of *B. burgdorferi* reads in datasets generated for specimens from Iowa, South Dakota, Nebraska and Kansas revealed heterogeneity in infection rates that correlated with geographic region of origin (Figure 6). For ticks from northeastern Nebraska (Thurston County) and Iowa, which consistently grouped with specimens from northern states in PCA‐ and admixture‐based analyses, 8 out of 11 specimens (72.7%) and 8 of 19 specimens (42.1%), respectively, had detectable *B. burgdorferi* reads that aligned across multiple contigs of the reference genome. Specimens from Kansas and South Dakota, despite their different regional affinities in previous analyses (southern vs. northern, respectively), showed no detectable *B. burgdorferi* infection (0 out of 5 specimens, each 0%). Ticks from east central Nebraska (Douglas, Sarpy and Dodge Counties), which demonstrate genomic admixture, showed no evidence of *B. burgdorferi* infection (0 out of 29 specimens, 0%). All read datasets show some degree of alignment to genomic contig 2 of the *B. burgdorferi* reference genome (Bol26) (Figure 6). Further investigation into specimens with reads aligned to genomic contig 2 only revealed these reads to align to a single ~900 bp region of this contig. Analysis using NCBI BLAST (Altschul et al. 1990) revealed that this region, and the reads aligning to it across all specimens, shared 100% nucleotide identity to a Rickettsial endosymbiont (GenBank: JQ031633.1) of *I. scapularis* ticks.

**FIGURE 6:**
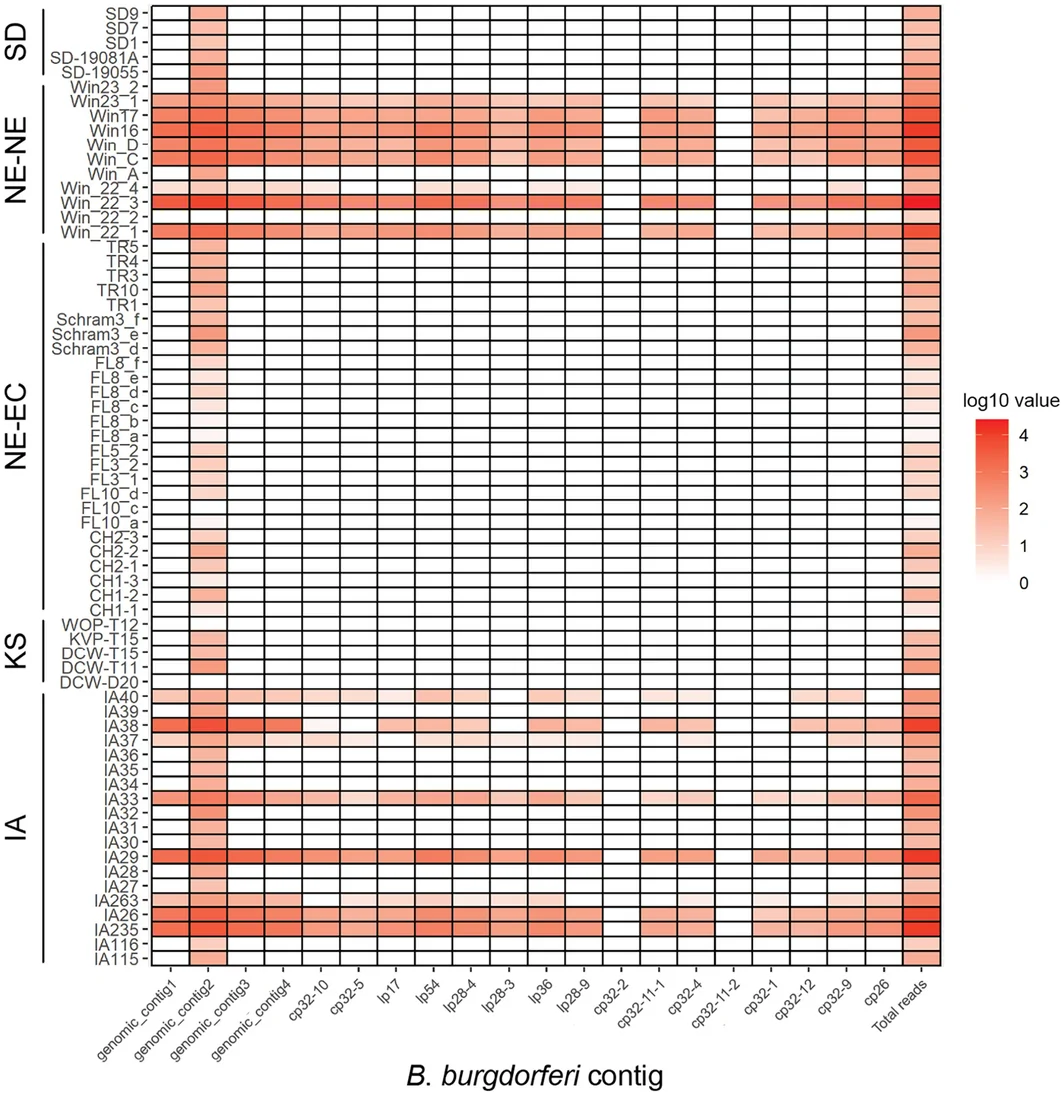
Heatmap of non‐ *Ixodes scapularis* reads that align to contigs of the *Borrelia burgdorferi* reference genome. Read data from individual ticks are shown as rows and are grouped by state of origin; *B. burgdorferi* contigs are shown as columns; plot segments are coloured by the number of reads aligned to each contig.

## 4 Discussion

In this study, we leveraged WGS data from individual *I. scapularis* ticks to describe genetic diversity, population structure, admixture and *B. burgdorferi* infection rates for recently established populations in the Great Plains region of the United States. Dramatically different *B. burgdorferi* infection rates reported for ticks collected in close proximity (< 90 miles) within Nebraska (Nebraska Tick Surveillance: Blacklegged Tick n.d.) led us to test the hypotheses that (1) Nebraska is a contact zone between historically isolated ancestral populations of *I. scapularis* and (2) strong north–south genetic differentiation and highly structured populations observed in the Eastern United States would also be present in recently established populations in the Great Plains. Accordingly, we generated 66 novel WGS datasets from individual ticks collected in Nebraska, South Dakota, Iowa and Kansas to compare to publicly available WGS data from ticks collected across much of the Eastern United States. Genomic data show that populations of *I. scapularis* on the westernmost edge of the species' distribution are structured by geography. Admixture occurs in a gradient across the Great Plains, and ticks collected from east central Nebraska (NE–EC) show the most distinct admixed ancestry, with nearly 50% of the genetic backgrounds of individual ticks being assigned to ancestral northern and southern populations. Presence of *B. burgdorferi* in ticks is easily identified with WGS data, and infection rates determined from these data recapitulate local surveillance data.

### 4.1 Sample Selection, Sequencing and Variant Calling

An objective of this study was to generate and make available WGS data from populations on the western‐most edge of the range of *I. scapularis* to provide a more complete picture of the population genomics of this species across the United States (Figure 1). Additionally, we aimed to determine the likely origin of recently established populations of *I. scapularis* in the Great Plains (South Dakota and Nebraska) which have not previously been included in population genomic studies. To put these data into appropriate context, we also generated WGS data from populations of *I. scapularis* in the neighbouring states of Iowa and Kansas. While recent studies of *I. scapularis* have included samples from Iowa (Frederick et al. 2023); Dong et al. 2025, this study represents the first WGS data from Iowa, South Dakota, Nebraska and Kansas. These datasets provide more comprehensive sampling of the range of *I. scapularis* by including populations in these zones of recent expansion. Additionally, we generated WGS data for ticks (*n* = 3) from the BEI laboratory colony. These specimens provide additional representation of Northeastern populations for this and future genomic studies, and help to characterize a commonly used laboratory resource. To ensure the accuracy of our results, we employed strict SNP filtration thresholds for the combined dataset of newly generated and publicly available data (*n* = 200 specimens). Our final LD‐filtered SNP dataset used in PCA‐ and admixture‐based analyses contained ~123,000 SNPs (see Table 1). This falls within the range of the numbers of SNPs analysed in recent population genomic studies of *I. scapularis* (e.g., from ~7000 SNPs in Frederick et al. 2023 and ~10,000 SNPs in Dong and Schoville 2026 to < 80,000 SNPs in Dong et al. 2025 and ~18–67 million in Schoville et al. 2024). It is worth noting that Schoville et al. imputed missing genotypes in their analysis, which is likely responsible for the greater number of SNPs included in their analyses. The results recovered here for population structure and genomic similarity within and among specimens represented by previously generated data were largely concordant with those of prior studies, supporting that a sufficient number of loci were sampled here.

### 4.2 Refined Approach for Purifying gDNA Across Sample Types

Sample storage and extraction methodology influenced the quantity of gDNA obtained from individual ticks (Figure S2), where adult ticks that were frozen prior to processing tended to produce higher gDNA yields. Given the substantially smaller size of nymphal ticks as compared to adults, generating quantities of gDNA sufficient for WGS can be difficult and has previously required strategies such as whole genome amplification (Dong et al. 2025). Modifying the Zymo Quick‐DNA HWM MagBead Kit protocol allowed us to obtain similar gDNA quantities from nymphal ticks and sets of eight legs removed from adult ticks. Though extractions from sets of legs were not prepared as sequencing libraries here, the quantities of gDNA obtained from leg sets were sufficient for input to most Illumina library preparation protocols, highlighting the utility of this approach for future studies where input tissue may be limited. Given that the Centers for Disease Control and Prevention (CDC) uses tick bodies for pathogen detection, but does not require legs, our results suggest that tick bodies can be sent to CDC for pathogen testing and their legs retained for generation of genomic data, resulting in reciprocally informative datasets and maximizing the scientific value of each tick collected. This further highlights the flexibility and utility of our modified Zymo Kit‐based extraction protocol, which has now been applied successfully to obtain gDNA sufficient for NGS from low‐input sample types across multiple species of parasites (Herzog et al. 2024).

### 4.3 Population Structure and Genetic Differentiation of *I. scapularis* in the Eastern United States

Aligned with previous studies, our results suggest strong geographic signal for population structure within *I. scapularis* across the Eastern United States (Xu et al. 2020; Sakamoto et al. 2014; Van Zee et al. 2013; Schoville et al. 2024; Frederick et al. 2023). Analyses based on PCA and admixture suggest WGS datasets are best‐described by two to four clusters. Division into two clusters, or assuming contribution from two ancestral populations, clearly distinguishes specimens from northern states in the Northeast and Upper Midwest from those from southern states. However, specimens from east central Nebraska were not easily distinguished as belonging to one of two clusters at *k* = 2 in the DAPC analysis, and strong genomic admixture was inferred for specimens from both east central Nebraska and Tennessee at *k* = 2 in admixture‐based analyses (see Figures 3A and 4). Schoville et al. also identified evidence of mixed genomic ancestry for specimens from Tennessee, with these specimens similarly falling between two major clusters representing northern and southern populations in their PCA (Schoville et al. 2024). Our PCA produced a classic horseshoe shape, which is well‐described in the population genomic literature as a result of IBD (Frichot et al. 2012; Novembre and Stephens 2008), and was also recovered by Schovillle et al. in their PCA of *I. scapularis* (Schoville et al. 2024). Results from *F*_ST_ estimations and visualization of IBD also support genomic distinction between populations in northern and southern states, with slightly elevated *F*_ST_ values inferred among southern populations and higher *F*_ST_ values inferred for north–south pairwise comparisons in nearly all instances (see Table 3; Figure 5). Low *R*‐squared values (< 0.2) for the plot of *F*_ST_ versus distance between sampling sites suggest that distance alone does not completely describe this distinction. This further supports the importance of a latitudinal gradient in separation: northern populations at great distances from one another could have relatively low *F*_ST_ values (e.g., Massachusetts versus Minnesota; ~1165 mi apart; *F*_ST_ 0.008) compared to northern versus southern populations that were closer in physical proximity but demonstrated greater genomic differentiation (e.g., Massachusetts versus Virginia; ~499 mi apart; *F*_ST_ 0.051). The range and pattern of pairwise *F*_ST_ values recovered here was similar compared to previous population genomic studies for *I. scapularis* . Guila‐Nuss et al., Frederick et al. and Schoville et al. each found relatively low *F*_ST_ values among northern populations, slightly elevated values among southern populations and higher values between the two regions (Schoville et al. 2024; Frederick et al. 2023; Gulia‐Nuss et al. 2016). This suggests little interbreeding between northern and southern populations, and slightly more isolation and population structure within southern states. At the higher *k*‐value tested here (*k* = 4), the Northeast and Upper Midwest (which included northeastern Nebraska and some specimens from South Dakota) were distinguished as separate populations, as was a group variously represented by specimens from more central states, including South Dakota, Iowa, east central Nebraska, Kansas and Tennessee (see Figure 3B). Previous population genomic studies identified degrees of structure for *I. scapularis* : both Schoville et al. and Dong et al. inferred *k* = 2–3 for their datasets (Schoville et al. 2024; Dong et al. 2025), though Guila‐Nuss et al. and Frederick et al. each inferred *k* = 5 (Frederick et al. 2023; Gulia‐Nuss et al. 2016). The five clusters of Guila‐Nuss et al. were represented by laboratory colony specimens, two southern populations (Florida vs. North Carolina and Virginia) and two northern populations (Massachusetts, Maine and Wisconsin versus New Hampshire and Indiana). The five clusters of Frederick et al. were best‐described as Northeastern, Upper Midwestern, Southeastern Atlantic and Southern Gulf, with central Florida identified as a distinct population. In all, this suggests a gradient of genomic differentiation across the range of *I. scapularis* , with a strong latitudinal signature for subpopulation structure. The two common laboratory strains for *I. scapularis* (BEI, established with ticks from Rhode Island; and OSU, established with ticks from Oklahoma) follow this pattern of latitudinal genomic differentiation, with BEI ticks recovered as more genetically similar to natural northern populations and OSU ticks recovered as more genetically similar to natural southern populations in each of our analyses (see Figures 2, 3, 4). Therefore, caution is warranted when attempting to draw conclusions about natural populations of *I. scapularis* across the species' geographic range based on experimental results as these two laboratory populations may also demonstrate differences in behaviour and host seeking previously identified in *I. scapularis* across its range. While differences in infection rates could be due to ecological differences, further investigation to understand if observed differences in infection rate in natural populations is associated with differences in *B. burgdorferi* susceptibility and transmission potential as well (Goddard et al. 2015). It is worth noting that our results demonstrated similar estimates of inbreeding coefficients for the two laboratory colony populations as compared to wild populations (Table 2), which is in line with laboratory colonies occasionally being supplemented with ticks from natural populations.

### 4.4 Western Populations of *I. scapularis* Demonstrate a Gradient of Genomic Admixture

By expanding the genomic data available from the western‐most edge of the range of *I. scapularis* , we were able to observe admixture occurring at different proportions across a north–south gradient throughout the Great Plains. Under *k* = 2 models, ticks from South Dakota, Nebraska and Kansas displayed evidence of genomic admixture between ancestral northern and southern populations, with the dominant contribution aligning with the latitudinal position of each state. For our DAPC analysis when *k* = 4 (Figure 3B), clusters also align with geography and consist of specimens from Northeastern, Upper Midwestern and southern states, with Cluster 3 as the exception. This cluster is made up of specimens that demonstrate substantial admixture irrespective of geography, as it includes South Dakota, Nebraska and Kansas, as well as Tennessee (see Figure 3B). Patterns of admixture within Nebraska are also latitudinally stratified. Ticks from northeast Nebraska were inferred to have genomes with predominantly ancestral‐northern contributions. Interestingly, ticks from eastern central Nebraska were inferred to have genomes with variable contributions from both northern and southern ancestral populations (see Figures 3 and 4). This was initially an unexpected result, as these two Nebraska subpopulations are separated by < 100 miles and occur in nearly identical ecoregions. However, other organisms have also been shown to demonstrate among‐lineage north–south genetic exchange within Nebraska, including striped skunks (Barton and Wisely 2012), deer mice (Pfeifer et al. 2018), Colorado potato beetles (Izzo et al. 2018) and sunflowers (Rieseberg et al. 1999), and strong genetic differentiation between populations of sickleweed has been documented in Nebraska within just 25 miles of separation (Piya et al. 2014). *Ixodes scapularis* has yet to be identified in southeast Nebraska. However, based on the pattern observed here, it seems likely that populations found in this region of the state will have genomic backgrounds that are also admixed, and—like ticks from Kansas—more similar to populations from southern states. As this study represents the first WGS data for ticks from the Great Plains, comparisons to prior studies cannot be made for this region. For Tennessee, which showed admixture proportions comparable to ticks from Kansas and has been included in prior studies, Schoville et al. similarly identified ticks from this state as admixed (Schoville et al. 2024). However, their demographic analysis suggests that admixture in Tennessee is historical rather than indicative of active gene flow between northern and southern populations. Although we did not conduct a demographic analysis in this study, the identification of genomic admixture in what are presumed to be more recently‐established populations of *I. scapularis* in the Great Plains may suggest recent contact between historically isolated northern and southern populations. Based on analysis of subpopulations within Nebraska, our results also suggest geographic distance alone is not sufficient to describe genomic differentiation within *I. scapularis* across the Eastern United States.

### 4.5 Identification of *B. burgdorferi* Reads From WGS Datasets

As expected, for each dataset generated in this study, the vast majority of reads (> 98%, Table S1) aligned to the *I. scapularis* genome. However, by aligning the remainder of the reads to the *B. burgdorferi* reference genome, we were able to infer *B. burgdorferi* infection status of *I. scapularis* ticks from WGS datasets alone (Figure 6). Presumed infection status largely aligns with public health surveillance data from each state (Hamik et al. 2023; Nebraska Tick Surveillance: Blacklegged Tick n.d.; Lingren et al. 2005; Oliver et al. 2017). Samples from Kansas and east central Nebraska did not show evidence of *B. burgdorferi* infection, where > 50% of ticks from Iowa and northeastern Nebraska had reads aligned across the *B. burgdorferi* genome. Given that *I. scapularis* ticks from South Dakota have previously been shown to be infected (Black et al. 2021; Maestas et al. 2018), we expected to identify reads aligning to *B. burgdorferi* from these ticks; however, our small sample size (*n* = 5) from South Dakota and a lower prevalence of infection from previous studies could explain why reads were not identified. The overall depth of coverage across the *B. burgdorferi* genome was low for any one sample, but the breadth of alignment across the nuclear genome and plasmids increases the reliability of using WGS data to determine infection status. The sequencing effort in this study was aimed at reaching ~10× depth of coverage; however, deeper sequencing of any *B. burgdorferi* positive sample likely would yield more complete genomes suitable for bacterial genomic analysis or phylogenetics. Of note, samples from east central Nebraska that display ~50% admixture from ancestral northern and southern populations have yet to test positive for *B. burgdorferi* through active surveillance or as a part of this study.

### 4.6 Study Limitations

Sample sizes for several populations included here were relatively low, represented by five or fewer specimens, including Rhode Island (BEI colony; *n* = 3), Oklahoma (OSU colony; *n* = 4), South Dakota (*n* = 5) and Minnesota (*n* = 2). Low sample sizes may have impacted comparative analyses and estimations of summary statistics for these populations. Notably, the Northeast and Lower Midwest regions were also not well represented in our sampling. Previously generated datasets available for these regions use reduced representation sequencing approaches (Frederick et al. 2023; Gulia‐Nuss et al. 2016), and were therefore not included in this study, although a recent analysis provided methodology to blend both WGS and reduced representation datasets (Dong and Schoville 2026). Additionally, we were unable to acquire *I. scapularis* samples from North Dakota where populations have recently established (Russart et al. 2014). Given our results suggest genomic admixture in latitudinally central states like Nebraska and Tennessee, future studies would benefit from inclusion of comparable WGS data from other potential zones of admixture, such as the Ohio River Valley within the Lower Midwest. Finally, it is worth noting that we have assumed all specimens included in analyses are conspecific (Oliver Jr et al. 1993). Should genomic and phenotypic divergence between populations in northern versus southern regions be deemed sufficient to once again recognize them as separate species (Spielman et al. 1979), analyses that specifically account for and identify genomic introgression may prove a more fitting approach.

## 5 Conclusions

In this study, we expanded the available WGS dataset for *I. scapularis* ticks to include samples from the western‐edge of the species' distribution, resulting in a more representative and complete picture of *I. scapularis* population structure in the Eastern United States. We have shown that there is a strong north–south signal for subpopulation structure in the Great Plains region and that admixture occurs across a gradient. Ticks from east central Nebraska demonstrate even admixture from ancestral northern and southern populations and do not show evidence of *B. burgdorferi* infection through WGS datasets or routine surveillance and pathogen testing, consistent with *I. scapularis* populations from the southern U.S. It is imperative to understand how genomic composition affects tick life history traits that can alter Lyme disease transmission risk, such as questing behaviour, host preference and *B. burgdorferi* vector competence. As *I. scapularis* populations continue to expand and previously isolated populations come into contact, future studies aimed at understanding the consequences of genomic admixture in *I. scapularis* ticks are warranted.

## Author Contributions

Research/Experimental Design: K.S.H., Z.P., R.C., J.R.F. Performed Research: K.S.H., Z.P., H.S., D.M., L.N., A.B., S.S.C., K.D., K.S., S.S., H.B., J.E.P., R.C.S., J.H., R.C., J.R.F. Analysed Data: K.S.H., Z.P., R.C., J.R.F. Writing/Editing Contribution: K.S.H., Z.P., H.S., D.M., L.N., A.B., S.S.C., K.D., K.S., S.S., H.B., J.E.P., R.C.S., J.H., R.C., J.R.F.

## Funding

This research was supported by the National Institute of General Medical Sciences (Grant P20GM103427), Nebraska Research Initiative (Grant 51385), Lyme Disease Association and the Centers for Disease Control and Prevention (Grant NU60OE000104).

## Conflicts of Interest

The authors declare no conflicts of interest.

## Supporting information


**Figure S1:** Schematic of bioinformatic pipeline used to generate, filter and analyse single nucleotide polymorphism (SNP) datasets for individuals of *Ixodes scapularis* .

**Figure S2:** Summary of DNA extraction efficiency from individual *Ixodes scapularis* sequenced as part of this study compared by amount of input tissue, preservation method and extraction method. The average mass of DNA purified from each individual using each method (Qiagen DNEasy Blood & Tissue Kit vs. Zymo Quick‐DNA HWM MagBead Kit) is provided in dark grey to the right of each box‐and‐whisker plot. The modified Zymo protocol performed comparably to the modified Qiagen protocol in terms of total average mass of DNA purified from an individual tick, especially considering the Zymo protocol was only used with nymphal ticks or the legs of ticks from a single adult while the Qiagen protocol was only used with whole adult ticks. Adult ticks collected from Iowa were outliers in terms of total mass of DNA purified from each individual.

**Figure S3:** Bayesian information criterion (BIC) and Akaike information criterion (AIC) scores versus cluster number for DAPC analysis. Lowest score for each criterion is indicated by a dotted line.

**Figure S4:** Demonstration of congruence between STRUCTURE and fastSTRUCTURE results for analysis of a reduced single nucleotide polymorphism (SNP) dataset representing a subset of individuals of *Ixodes scapularis* from the Great Plains newly‐sequenced as part of this study. (A) Admixture plots for STRUCTURE (left) and fastSTRUCTURE (right); (B) STRUCTURE Harvester *k*‐value estimation output and (C, D) fastSTRUCTURE *k*‐value estimation output. Each sampling site within Nebraska is labelled by region (northeastern Nebraska, NE–NE; or east central Nebraska, NE‐EC).

**Figure S5:** mec70577‐sup‐0005‐FigureS5.png. *k*‐value estimation results for fastSTRUCTURE (A, B) and ADMIXTURE (C). Based on the complete *Ixodes scapularis* single nucleotide polymorphism (SNP) dataset (123,186 SNPs; *n* = 200 individuals).

**Table S1:** Metadata for all tick samples included in this study.

## Data Availability

WGS from individual specimens is available on the European Nucleotide Archive (ENA) under study accession ERP194443 and is cross‐listed to NCBI Sequence Read Archive (SRA) under the BioProject Accession PRJEB114136. Metadata for each specimen is available in Table S1 including all accession numbers. All code used to conduct analysis and generate figures is available on our GitHub: https://github.com/ZachPella/United.states.WGS‐blacklegged‐tick‐full‐pipeline.
